# Impact of an Electronic Monitoring Intervention to Improve Adherence to Inhaled Medication in Patients with Asthma and Chronic Obstructive Pulmonary Disease: Study Protocol for a Randomized Controlled Trial

**DOI:** 10.2196/resprot.7522

**Published:** 2017-10-23

**Authors:** Claudia Gregoriano, Thomas Dieterle, Selina Dürr, Isabelle Arnet, Kurt E Hersberger, Jörg D Leuppi

**Affiliations:** ^1^ University Clinic of Medicine Cantonal Hospital Baselland Liestal Switzerland; ^2^ Department of Pharmaceutical Sciences University of Basel Basel Switzerland; ^3^ Faculty of Medicine University of Basel Basel Switzerland

**Keywords:** asthma, pulmonary disease, chronic obstructive, medication adherence, randomized controlled trial

## Abstract

**Background:**

Despite progress in pharmacological and non-pharmacological treatment in recent years, the burden of disease among patients with asthma and chronic obstructive pulmonary disease (COPD) is high and patients are frequently hospitalized due to exacerbations. Reasons for uncontrolled diseases are manifold, but are often associated with poor inhalation technique and non-adherence to the prescribed treatment plan. This causes substantial mortality, morbidity, and costs to the healthcare system. In this respect, the study of causes for non-adherence and the development of measures to increase and maintain treatment adherence in chronic diseases is of major clinical importance.

**Objective:**

The primary objective of this study is to investigate the impact of using specific, validated electronic devices on adherence to inhaled medication in patients with chronic obstructive lung diseases such as asthma and COPD. Furthermore, it aims to assess the impact of a reminder and close supervision of the course of disease and quality of life.

**Methods:**

In this ongoing prospective, single-blind, randomized controlled study, adherence to inhaled medication is analyzed over a 6-month period in at least 154 in- and outpatients with asthma or COPD who have experienced at least 1 exacerbation during the last year. Adherence is measured using electronic data capture devices, which save the date and time of each inhalative device actuation and transfer these data daily via a wireless connection to a Web-based database. Patients are randomly assigned to either the intervention or the control group. The clinical intervention consists of an automated and personal reminder. The intervention group receives an audio reminder and support calls in case medication has not been taken as prescribed or if rescue medication is used more frequently than pre-specified in the study protocol. During the study, participants are assessed every 2 months in the form of clinical visits.

**Results:**

Recruitment started in January 2014. To date, a total of 169 patients have been recruited. Follow-up assessments are still ongoing. The study will be concluded in the first quarter of 2017. Data analysis will take place during 2017.

**Conclusions:**

Few studies have investigated medication adherence in patients with chronic obstructive lung diseases. With this prospective study design and the use of state-of-the-art devices for measuring adherence, we expect scientifically relevant and clinically meaningful results that will have a substantial and positive impact on the provision of healthcare in chronically ill patients suffering from asthma or COPD.

**Trial Registration:**

ClinicalTrials.gov: NCT02386722; https://clinicaltrials.gov/ct2/show/NCT02386722 (Archived by WebCite at http://www.webcitation.org/6oJq1fel0)

## Introduction

Asthma and chronic obstructive pulmonary disease (COPD) are highly prevalent lung diseases requiring daily and often lifelong use of inhaled medication [1]. According to the World Health Organization (WHO), COPD currently represents the fourth leading cause of death worldwide and is predicted to become the third leading cause of death by 2030 [2]. The prevalence of COPD is increasing due to continuing exposure to COPD risk factors (e.g. tobacco smoke or air pollution) and the continuously aging world population) [3]. The prevalence of asthma is increasing as well [4]. In Swiss adults, the prevalence of asthma and COPD was found to be around 7% and 7% to 9%, respectively [5,6].

### Treatment and Disease Control

Despite progress in pharmacological and non-pharmacological treatment in recent years, the burden of disease imposed by asthma and COPD remains high and patients may be frequently hospitalized due to exacerbation. Based on data from the Swiss COPD Cohort Study, COPD exacerbation rates are high at 23% per year [7]. Acute exacerbations are a risk factor for disease progression and are associated with increased mortality [8]. A survey published by Leuppi et al showed that the level of asthma control in Switzerland is very low with 15% of the investigated patients [9]. This has also been confirmed by a cross-sectional survey by Miedinger et al who found controlled asthma in 27% of all patients according to the international Global Initiative for Asthma (GINA) guidelines [10]. However, good adherence to therapy can increase the likelihood of achieving better disease control [11].

Reasons for insufficient disease control in asthma and COPD patients are manifold. They are frequently associated with poor inhalation technique and non-adherence to prescribed treatment plans, which may influence mortality and morbidity and pose a financial burden on healthcare systems [12].

### Medication Adherence

According to the WHO, adherence is defined as “the extent to which a person’s behavior (including medication-taking) corresponds with agreed recommendations from a healthcare provider” [13]. Adherence represents the basis for effective drug therapy and complete disease control. It is a multidimensional issue with several influencing factors. The WHO classifies these factors into 5 dimensions: socioeconomic-related factors, healthcare team and system-related factors, condition-related factors, therapy-related factors, and patient-related factors [13]. Furthermore, 2 different patterns of non-adherence behaviors are observed in patients, namely intentional and unintentional non-adherence. Intentional non-adherence describes the deliberate discontinuation or reduction of the intake of medication in case of absence of symptoms [14], which may be due to a lack of understanding of the disease course and treatment aims. In addition, the occurrence of side effects can also lead to intentional non-adherence. Unintentional non-adherence, however, is observed when patients do not follow treatment plans due to reasons out of their control, such as forgetfulness, cognitive impairment, or physical disability [15]. In patients taking inhaled medication, impaired vision or musculoskeletal disorders can affect their ability to use the inhaler devices correctly [16]. Other reasons for unintentional non-adherence are complex medication regimes, poly-pharmacy, and the use of multiple inhalers [17,18]. Non-adherence not only leads to suboptimal treatment of individual patients, but may also cause disease prolongation and increased hospital readmission. Finally, it can increase costs for the healthcare system [19].

Based on a systematic literature review of medication adherence literature, Vrijens et al proposed a new taxonomy for describing and defining adherence to medication [20]. The Ascertaining Barriers to Compliance (ABC) taxonomy considers a sequence of events that have to occur for a patient to achieve an optimal benefit from their prescribed treatment regimen and to minimize the risk of harm. This process is divided into 3 essential components: initiation, implementation, and persistence. The process starts with initiation characterized by the intake of the first dose of a prescribed medication. It continues with implementation of the dosing regimen, which is defined as the extent to which a patient’s actual dosing corresponds to the prescribed medication during the time period from initiation to the last dose taken. The last step of the process is persistence, which refers to the time from initiation to eventual discontinuation. After discontinuation, a period of non-persistence may follow until the end of the prescription period.

As such, non-adherence to medications can occur in the following situations: late or non-initiation of a prescribed treatment, suboptimal implementation of the dosing regimen, or early discontinuation of the treatment. This classification is particularly helpful in framing focused research questions as well as finding measures and data to answer them.

Adherence to long-term therapy is estimated to be around 50%, as shown in a systematic review summarizing the results of randomized controlled trials (RCTs). It investigated interventions in order to help patients follow prescriptions for medications [21]. Among patients with asthma, rates of non-adherence ranged from 30% to 70% [22]. Levels of non-adherence are comparably high in patients with COPD, ranging from 43% to 58% [23,24]. Adherence to medication can be measured using direct or indirect methods. Direct methods encompass direct observation of drug intake or measurement of drug concentration, such as markers in the blood, urine, or other body fluids. Indirect methods include assessment of a patient’s clinical response, pill count, rates of refilling prescriptions, patients’ self-report, or the use of electronic monitoring devices [25,26]. While none of these methods are currently considered the gold standard for measuring adherence to medications [27,28], the emerging method of choice is electronic monitoring devices [29].

Self-reporting by patients was shown to be the most cost-effective approach to the assessment of adherence in clinical and research settings [30]. However, being a subjective method, it also bears the highest risk of overestimating adherence compared to electronic measurements [31].

Observational retrospective studies based on dispensing data from pharmacy record databases analyzed refill adherence for different inhaled medication in patients with asthma and COPD [32-34]. The importance of refill adherence is limited, since this measurement cannot assess the timing of the ingested or inhaled doses that depend on the duration of drug action, which in turn has an important impact on the efficacy of treatment [35].

To investigate the variability in timing and medication adherence, measurements of dose and timing are necessary, which can be done with electronic medication monitors. Electronic monitoring provides precise data on timing and the pattern of inhaler actuation. In addition, it may detect multiple successive actuations (dumping) [36].

Electronic monitoring methods such as SmartInhaler devices (Adherium Ltd., Auckland, New Zealand) are non-invasive and represent one of the best ways to detect adherence patterns when using additional tools attached on the inhaler devices [37]. SmartInhaler devices have been validated for the assessment of adherence to inhaled medication on a daily basis [38]. They are able to track the time and date of each actuation of the inhaler device (incorporated switch activates by depression or rotation of the device) and transmit the data via a wireless connection to a secure Web database [38]. SmartInhaler devices have been used in several studies measuring adherence to inhaled medication [39,40]. In a study on patients with asthma using inhaled corticosteroids, the integrated audio-visual reminder function of these devices significantly improved adherence to inhaled medication [41].

Adherence to orally administered drugs or inhaled medications available, such as powder capsules, can be measured by applying a novel technology called Polymedication Electronic Monitoring System (POEMS). This technology is composed of a printed, self-adhesive polymer film carrying loops of conductive wires that can be affixed to multidose punch cards (Pharmis GmbH, Beinwil am See, Switzerland) with 28 cavities. Every time a powder capsule is taken out of the blister, a loop is broken leading to changes in electrical resistance that can be measured and recorded with date and time [42]. The reports generated by SmartInhalers and POEMS detect whether the patients have taken the medication at the right time and dose.

### Interventions to Improve Medication Adherence

Maintenance of sufficient adherence to the prescribed medication is a critical factor in achieving therapeutic success, particularly in chronic diseases. Haynes et al [43] reviewed randomized controlled intervention trials to improve adherence to pharmacological regimens in patients with chronic diseases, including asthma. Both adherence and clinical outcomes were measured in these studies. The authors found that less than 50% of the interventions achieved a significant improvement of adherence while only 30% demonstrated an improvement in clinical outcome. The greatest success was attained with complex interventions combining several strategies (information, reminders, self-monitoring, reinforcement, counseling, telephone follow-up, supportive care, etc). [43]. Lu et al [44] showed that disease management interventions are associated with short- and long-term improvements with regards to the process and quality of care; in particular, when using structured, population-based and multidisciplinary approaches for the identification, treatment, and monitoring of patients with chronic illness. This review also suggested that coordinating pharmacist services as a component of the process of care can improve quality of life, medication adherence, and clinical outcomes in chronic patients [44]. However, particularly successful intervention components could not be determined specifically [45].

### Study Objectives

The objectives of this study are (1) to investigate the impact of using specific, validated electronic devices on adherence to inhaled medication in patients with asthma and COPD; and (2) to assess the effect of an acoustic reminder and close supervision on the course of disease and quality of life.

## Methods

### Participants and Recruitment

In- and outpatients with a diagnosis of asthma bronchiale or COPD from several hospitals in the Basel region and patients treated by pulmonologists in private practice are screened for eligibility (Table 1). Advertisements are distributed in the form of posters, flyers, as well as on ad-screens (Cantonal Hospital Baselland Liestal and Bruderholz), communicating the most important information about the study. Advertisements are also placed in local newspapers.

**Table 1:** Recruitment locations and related recruitment types.

Hospital	Location	Recruitment
Cantonal Hospital Baselland	Liestal, Switzerland	Screening of hospitalized patients
		Screening of the emergency department
		Screening of DRG^a^ lists
Cantonal Hospital Baselland	Bruderholz, Switzerland	Screening of DRG^a^ lists
		Collaboration with the pulmonology department
Claraspital	Basel, Switzerland	Collaboration with the pulmonology department
Clinic Barmelweid	Barmelweid, Switzerland	Collaboration with pulmonology department
Gesundheitszentrum Fricktal AG	Rheinfelden, Switzerland	Collaboration with the pulmonology department

^a^DRG: diagnosis related group.

Initially, inclusion and exclusion criteria are checked via telephone, during hospitalizations, or practice visits. Eligible patients are invited for an introductory training course. Before the start of the study, the investigator provides written and verbal information about content and duration of the study. The investigator obtains written consent from patients confirming their willingness to participate in the study.

### Inclusion and Exclusion Criteria

The study inclusion and exclusion criteria for male and female participants are shown in Textbox 1 [46]. Enrolment started January 2014 and will end when at least 154 individuals are included in the study.

**Inclusion and exclusion criteria.**

Criteria

Inclusion

Aged 18 years or older

Have an established asthma-diagnosis according to the Global Initiative for Asthma (GINA) guidelines and/or

- Have an established COPD diagnosis according to the Global Initiative for Chronic Obstructive Lung Disease (GOLD) guidelines (severity GOLD I-IV based on the international GOLD-Criteria) [46] and
- Are prescribed daily inhaled medication (controller medication for a daily maintenance treatment)
- Had at least one exacerbation in the previous 12 months before study start

Exclusion

- Suffering from malignancies and/or other severe diseases
- Insufficient in the German language
- Pregnant or lactating

### Study Design and Procedures

**Figure 1:**
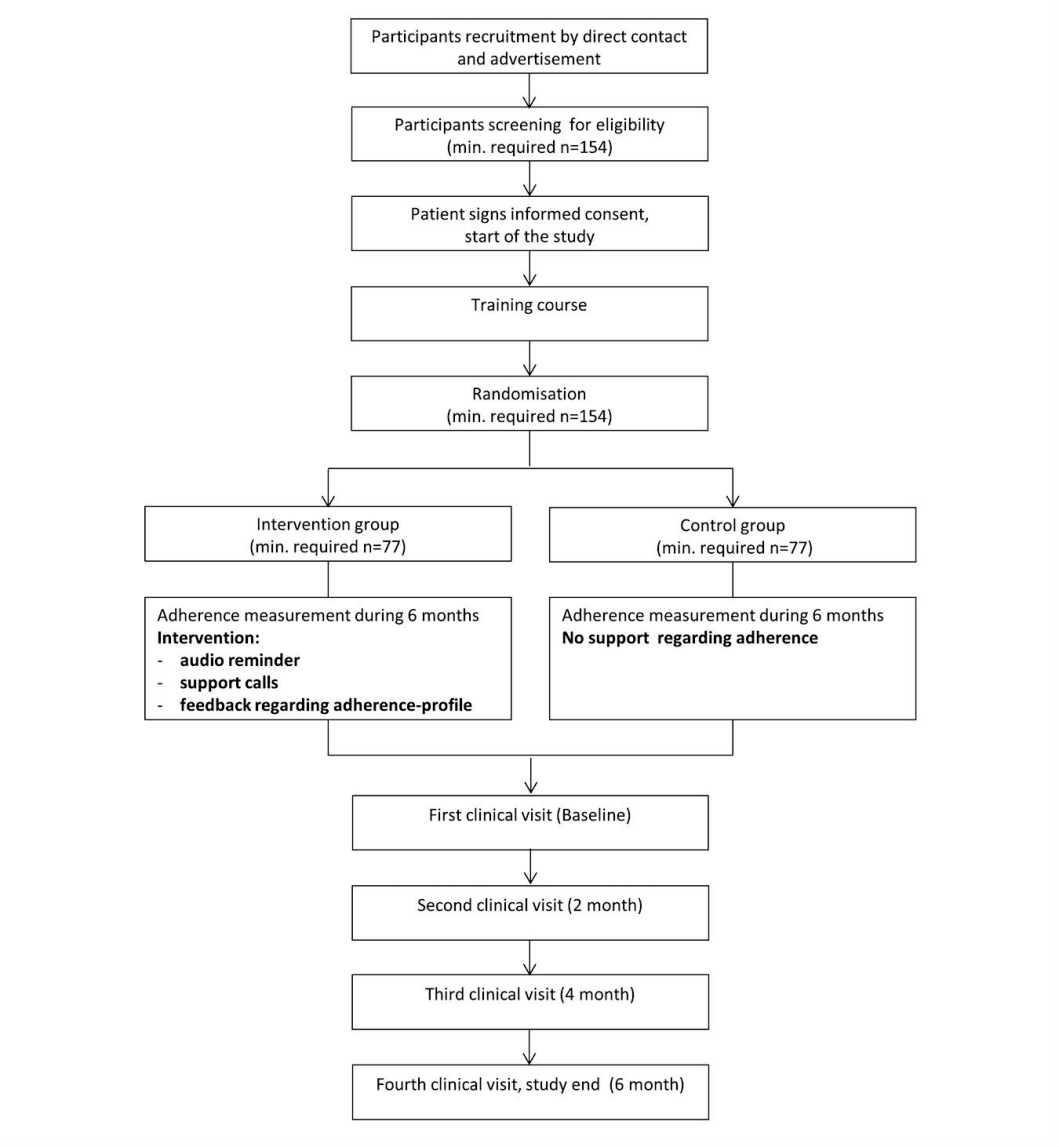
Study flow chart based on the Consolidated Standards of Reporting Trials (CONSORT) guidelines.

In this prospective, single-blinded RCT, 169 participants are followed for up to 6 months (Figure 1). Prior to study start, patients have to be in a stable phase of their obstructive lung disease. This is defined as an exacerbation-free period of at least 1 month prior to commencement of the study and no current hospitalization for any other medical condition. Study participants will continue to be cared by their usual treating physician(s) who decide on all prescriptions and treatments.

All participants take part in a training course before the baseline visit, which takes approximately 45 to 60 minutes to complete. The goal of the training course is to provide refresher training on inhalation techniques in order to ensure that all participants are at the same level of disease knowledge and use their medication correctly. The training begins with a brief introduction about asthma and COPD. Afterwards, the most frequently used devices are presented and briefly demonstrated. Correct technique depends on inhaler type and it is important that patients use their own inhaler correctly. Common mistakes and problems associated with the use of the devices are explained. The correct use of the individual devices is demonstrated in a short film (produced by the “Deutsche Atemwegsliga” Bad Lippspringe, Germany) [47], which presents the most important steps to follow in order to achieve an effective inhalation. Notably, it has been shown that the manufacturer’s instruction sheet is not effective enough to achieve correct techniques [48-50]. However, the combination of verbal and visual instructions seems to have a higher success rate in improving the application of inhaler devices [51]. At the end of the training, participants are given the opportunity to ask questions concerning the devices.

Visits take place at baseline (T0), after 2 (T1), 4 (T2), and 6 months (T3) and will take between 45 to 60 minutes, depending on the patient, regardless of the group they belong to. Each visit includes a spirometry test (EasyOne Pro, ndd Medizintechnik AG, Zurich, Switzerland), measurement of diffusion capacity (EasyOne Pro, ndd Medizintechnik AG, Zurich, Switzerland), and exhaled nitric oxide (NIOX MINO, Aerocrine AB, Sweden) and carbon monoxide (piCO^+^Smokerlyzer, Bedfont Scientific Ltd., Kent, UK) levels. To detect false device applications, each patient is asked to demonstrate the inhalation technique with all prescribed devices to the investigator by using a placebo device (to avoid overdosing). Moreover, participants have to complete the COPD Assessment Test (CAT) [52], the Asthma Control Test (ACT) [53], the St. George’s Respiratory Questionnaire (SGRQ), and the Short Form (SF)-36 [54,55] to assess quality of life at baseline, after 2, 4, and 6 months. To investigate patients’ beliefs about the necessity of the prescribed medication as well as their concerns about the potential adverse consequences of taking it, the Beliefs About Medicines Questionnaire (BMQ) is used at baseline [56,57]. Throughout the 4 visits, information about exacerbations since the last visit are also obtained.

### Randomization

Participants are randomly assigned either to the intervention or to the control group. The intervention group is provided with an acoustic reminder for inhalation and receives support calls when the medication is not taken as prescribed. The control group does not receive further support regarding their adherence. A randomization list with study group allocation is generated using R (RStudio, Boston, US). The randomization procedure is provided in a block size of 2. Therefore, examinations between study groups are sequent. This reduces the risk of a season effect between the 2 study groups. Furthermore, the patients are not aware of which group they have been randomized to (single-blinded).

### Clinical Intervention

The clinical intervention consists of an automated and personal reminder. Patients assigned to the intervention group receive an audio-reminder, generated by a mobile phone with app capabilities (smartphone). For patients with SmartInhaler, the inhalation times are entered on the Smartinhalerlive website by the investigator. These are then generated by an app directly onto the participant’s mobile phone. For patients using POEMS, the inhalation times are entered by the investigator directly in form of an alarm clock onto the mobile phone. Patients are allowed to choose the inhalation times themselves, depending on their personal habits and daily routine. This allows for times during the workday and weekend to be defined. Since the inhalation actuation does not stop the device alarms, the reminder generated by the SmartInhaler app and those generated by the mobile phone have to be quitted by the patients themselves. Moreover, these patients receive support calls carried out by the pharmacist when the use of rescue medication doubles or if the inhaled medication is not inhaled as prescribed for more than 2 consecutive days. In exceptional cases and in the absence of the pharmacist, the support calls are carried out by the responsible study nurse who has been trained accordingly. Participants also receive feedback from the pharmacist on their adherence at each visit, especially for the results of the POEMS.

Patients assigned to the control group have no reminder and will receive no further support regarding their adherence to their inhaled medication.

### Sample Size

Power calculation is based on “time to next exacerbation”. A previous study has shown that 30% of patients with COPD are readmitted within 6 months because of an exacerbation [58]. Exacerbation rate could be reduced by 30% with an educational program [59]. Since our intervention is not only based on an educational program but on close supervision during the study period, we expect a bigger effect of our intervention, resulting in an assumed endpoint reduction of 40% (12/30), with 11% (8/70) of patients experiencing an exacerbation in the intervention group. This corresponds to a hazard ratio (HR; intervention/control) of 0.36, taking into consideration the time-to-event-curve for the primary outcome (time to next exacerbation). Assuming a sample size of 70 participants for each study group, there is a power of 80% to detect a HR of 0.36 based on a 1-tailed test, since only a decrease of the exacerbation risk is of interest and expected. The calculation is based on the assumptions mentioned above and on a 1-tailed test with a significance level of 5%. Furthermore, 14 additional participants (7 for each study group) will be added to account for dropouts. Therefore, a total of 154 participants will be included in this study.

### Measurement of Objective Adherence

In both groups, adherence is measured using SmartInhalers and POEMS as outlined above. Daily measurements are started after the baseline visit (T0) and are continued until the end of the study (T3). All participants are aware that their adherence is measured during the whole study period using the delivered devices. Hence, a possible “hawthorne effect” can result, which represents a change in patient’s behavior as a consequence of being monitored during a study [60]. However, previous studies showed that there is no better adherence in patients who were informed that their drug intake was being monitored compared to those patients who were unaware of the monitoring [61,62].

Recorded data are uploaded daily at 00:00 to a Web-based database via a wireless connection. Participants are asked to take their medication at the first visit in order to ensure the correct handling and usage of the SmartInhaler. Once the devices are installed on the inhalers, patients can use their medication as usual.

Currently, no monitoring devices exist that are specifically developed for monitoring the adherence of the newly introduced inhalation-device Ellipta. To assess adherence in patients undergoing treatment with Ellipta, a SmartInhaler with a placebo-device is handed out and patients are instructed to trigger a puff of the placebo every time when they inhale their active treatment. This procedure allows an indirect recording of date and time actuation of the Ellipta inhaler.

POEMS are used for inhalation with powder capsules (Breezhaler and HandiHaler). The capsules are pre-filled for the following 2 weeks with a patient’s individualized prescription plan (mostly one time daily inhalation of capsule contents). The multidose punch cards are filled manually by a pharmacist. Participants who apply Breezhaler and HandiHaler will receive 1 multidose punch card for every 2 weeks. Every time the patients break a loop for taking the capsules, the date and time are recorded on a microchip, which can be read out when patients bring back the empty punch card.

### Data Collection and Outcome Measures

The primary outcome of this study is “time to next asthma or COPD exacerbation”, defined as acute-onset worsening of the patient’s condition beyond day-to-day variations requiring interaction with a health care provider [63]. Outcome is expressed as the number of exacerbations since the last visit with the exact period of exacerbation as well as the number of exacerbations followed by hospitalization. If patients are not able to provide information about the time of exacerbation, the treating physician will be contacted.

Sociodemographic variables such as gender, civil status, age, educational level, and employment status are obtained by a generic questionnaire during the baseline visit. Furthermore, smoking status is assessed from medical history and expressed as pack years (py; number of smoking years times the number of smoked packs per day). Body height and weight are signified by body mass index (BMI; body weight/[body height]^2^). In addition, disease-related questions such as allergies, comorbidities, current medication, and number of exacerbations in the previous 12 months are recorded, including hospitalizations and emergency department attendance.

This project focuses on the implementation of a prescribed dosing regimen. Objective adherence will be analyzed according to the definitions shown in Textbox 2 [64].

**Objective adherence definitions.**

Definition

- Taking adherence: (number of puffs inhaled during 24 hours/number of puffs prescribed during 24 hours) x 100
- Timing adherence: (number of correct dosing intervals during 24 hours/number of dosing intervals during 24 hours) x 100; correct dosing intervals are prescribed intervals ± 25%:
  - For once daily dosing: 24 hours ± 25% = 18 hours to 30 hours
  - For twice daily dosing: 12 hours ± 25% = 9 hours to 15 hours
  - For three daily dosing: 8 hours ± 25 % = 6 hours to 10 hours
- Gaps: (number of days without inhalation during the whole study period/number of days in same time period) x 100
- Maximal gap length: number of consecutive days of the longest period of time without inhalation

Throughout all visits, the following lung function tests are performed to assess changes in lung function: spirometry (FEV_1_, FVC, FEV_1_/ FVC), diffusion capacity, and nitric oxide and carbon monoxide measurements.

During each visit (T0 to T3), participants are asked to demonstrate how they actually use their device at home to evaluate the inhalation technique. For this purpose, placebo devices are used to prevent overdosing. Correctness of inhaler use is assessed using pre-defined checklists for each inhaler type based on user guidelines and instruction package inserts from the manufacturers [65-70]. Correct inhaler usage is defined as correct performance of every step on the checklist. Incorrect inhaler usage is defined as 1 or more steps done incorrectly. A total score is calculated with 0 (incorrect application) and 1 (correct application) and applied to every step. Possible errors are corrected by verbal instruction and visual demonstration. For ethical reasons the correction was performed in both groups. Patients demonstrate their inhalation technique until it is performed correctly.

At baseline, the BMQ is used to assess patients’ beliefs about the need of the prescribed medication and their concerns about the potential adverse consequences of taking it.

Changes in quality of life are investigated at baseline, after 2, 4, and 6 months using different disease-specific questionnaires: SGRQ, CAT, and ACT. To determine general quality of life, the SF-36-questionnaire is used.

Data collection will end as soon as all study participants have finished the 6-month observational period and have had the fourth clinical visit.

### Statistical Analysis

Statistical analyses, including descriptive statistic and survival analyses, are carried out using the software R (RStudio, Boston, US) and SPSS (IBM Corporation, Armonk, US). Statistical significance is set at the 5% level. Time to next exacerbation is compared by applying the Kaplan-Meier method and Cox proportional hazard model. Results will be reported as a HR with a corresponding confidence interval (CI) of 95 % and *P* values. A HR smaller than 1 is expected. This implies that the intervention group will have a smaller risk for exacerbations. Associations between time to between exacerbation and independent predictors will be analyzed (taking adherence, timing adherence, and gaps without inhalation). Comparisons of secondary parameters are done using *t* tests or chi-square tests (or their nonparametric equivalents if data are not normally distributed).

### Missing Data and Dropouts

Patients will be rated as dropout when they are excluded from the study at their own request or if they are no longer able to participate in the study until the final visit. Patients who are not able to undergo all clinical examination during the follow-up visits will remain in the study. Multiple imputation methods will be used to impute missing data with less than 25% missing values. This is typically more efficient than complete case analysis when covariates have missing values [71].

### Ethics and Dissemination

This study is conducted according to the Helsinki Declaration and according to the good clinical practice guidelines. Study participation is voluntary and can be revoked at any time without specification of reasons and will have no disadvantages for their future medical care. The study was approved by the Ethics Committee Northwest/Central Switzerland (registry number: EK-269/13) and was registered with Clincialtrials.gov (NCT02386722). In case of any considerable deviations from the actual study protocol, the investigator will send an amendment for further approval from the ethical committees. The results of this study will be disseminated via seminar, conference presentations, and academic, peer-reviewed journals.

### Data Security and Disclosure of Original Documents

Patient data are collected and stored under confidentiality rules. For reports, data collection, and administrative forms an anonymization will be done and participants will be assigned a study identification (ID) (PXXX). All study-related data and documents are stored on a protected server of the Cantonal Hospital Baselland. Data access is limited to members of the medical research group at the Cantonal Hospital Liestal. After study completion, all documents and informed consent forms will be retained in the archives of the University Department of Internal Medicine at the Cantonal Hospital Liestal for 10 years according to applicable Swiss regulatory requirements.

## Results

This is a single-centre, randomized controlled study. It is performed at the Cantonal Hospital Baselland, Liestal, and Bruderholz, Switzerland. Recruitment started in January 2014, and to date, a total of 169 patients have been recruited. Follow-up assessments are still ongoing. The study will be concluded in the first quarter of 2017. Data analysis will take place during 2017.

## Discussion

To date, only a few studies have investigated medication adherence in patients with chronic obstructive lung diseases. These studies were retrospectively analyzed, limited to refill adherence, and had several important limitations such as the lack of assessment of the relationship between the duration of drug action and the timing of the ingested doses, which impacts the efficacy of treatment [15]. Other disadvantages of this measurement are missing data when refills were obtained outside of the investigated system and incomplete records if the medication plan is verbally modified by the prescriber without informing the dispensing pharmacy. Moreover, assumptions have to be made on medication intake behavior, if it is taken according to the prescription, and corresponds to the prescribed refilling [72].

We expect that a regular adherence reminder and close supervision by a healthcare professional will have a beneficial effect on adherence to inhaled medication in patients with asthma or COPD, resulting in an increased time to next exacerbation. In addition, we assume that improved adherence will increase the quality of life of these patients.

With the prospective study design and the use of state-of-the-art devices for measuring adherence, we expect scientifically relevant and clinically meaningful results that will have a substantial and positive impact on the provision of healthcare in chronically ill patients suffering from asthma or COPD.

## Abbreviations

ACT: Asthma Control Test
BMQ: Beliefs about Medicines Questionnaire
CAT: COPD Assessment Test
CI: confidence interval
COPD: chronic obstructive pulmonary disease
HR: hazard ratio
POEMS: Polymedication Electronic Monitoring System
RCT: randomized controlled trial
SF-36: Short Form 36
SGRQ: St. George’s Respiratory Questionnaire
WHO: World Health Organization
